# The integrin odyssey – a journey full of fundamental discoveries

**DOI:** 10.1242/jcs.263999

**Published:** 2025-09-24

**Authors:** Reinhard Fässler, Arnoud Sonnenberg

**Affiliations:** ^1^ Max Planck Institute of Biochemistry, 82152 Martinsried, Germany; ^2^ Technical University Munich, 85748 Garching, Munich, Germany; ^3^ The Netherlands Cancer Institute, Division of Cell Biology, 1066CX Amsterdam, The Netherlands

**Keywords:** Integrin, Focal adhesion, Conformational transitions, Signalling, Adhesome

## Abstract

Integrins are widely expressed cell adhesion receptors that link extracellular matrix proteins to the cytoskeleton. Most integrins connect to the actin cytoskeleton through different types of adhesion plaques, including nascent adhesions and focal adhesions. They regulate adhesion strength in response to the mechanical force exerted by actomyosin across the plasma membrane. Beyond serving as mechanical connectors, integrins also play a crucial role in regulating cellular behaviour through biochemical signalling pathways. Given their essential functions, integrins are vital for the survival of metazoans and are important therapeutic targets for treating various human diseases. This Perspective highlights the work that led to the discovery of integrins and the principles of how they function.

## Teaser

**Summary:** This Perspective highlights the key discoveries in integrin research that laid the foundation for a successful field.

## Introduction

Our entry into the integrin field was serendipitous. Reinhard entered the field in the early 1990s. Before this, he had completed his training as clinician and joined the laboratory of Rudolf Jaenisch at the Whitehead Institute (Cambridge, MA, USA) from the late 1980s to the early 1990s as a postdoctoral fellow. His aim was to learn how to use gene targeting technology by analysing collagen-encoding genes in mice. A year after his arrival at the Whitehead Institute, he was asked to help to establish embryonic stem cell technology in the laboratory of Richard Hynes, which was located across the street (Massachusetts Institute of Technology, MA, USA). Hynes and co-workers strived to generate germline mice carrying a disrupted fibronectin gene. Reinhard visited the Hynes lab regularly over several weeks, showing Elizabeth George how to handle and manipulate embryonic stem cells. In return, he was invited to join the progress report lectures presented at an ‘integrin club’, a regular assembly of integrin aficionados of the Boston area. The interesting discussions at these meetings in this emerging field inspired him – despite warnings – to shift his focus from studying ‘collagens’ to analysing the β1 integrin (*ITGB1*) gene in mice. This decision marked the beginning of his independent scientific career in 1992 as a young principal investigator at the Max Planck Institute of Biochemistry in Martinsried, Germany.

Arnoud joined the laboratory of Renato Dulbecco at the Salk Institute (San Diego, CA, USA), in 1980 to learn how to use hybridoma technology. Upon returning to the Netherlands, he produced a series of monoclonal antibodies (mAbs) against mouse mammary tumour antigens, which he used to define cell differentiation lineages of the mouse mammary gland. Among these antibodies was GoH3, whose antigen co-distributed with laminin in histochemical analyses. Subsequent studies identified the GoH3-defined antigen as platelet glycoprotein (GP) Ic, which forms a complex with GPIIa, and established the GPIc/IIa complex as a new member of the very late antigen (VLA) protein family (also known as the β1 integrins), called VLA6 (α6β1), that binds laminin. After this discovery, and the identification that the α6 subunit can pair with an alternative β subunit, β4 integrin, Arnoud focused his research on this novel α6β4 integrin complex. The α6β4 integrin is expressed on epithelial cells, forms an important component of hemidesmosomes (junctions that mediate stable adhesion of epithelia to basement membranes) and, unlike most other integrins, associates with the intermediate filaments rather than the actin filament system.

**Fig. 1.**
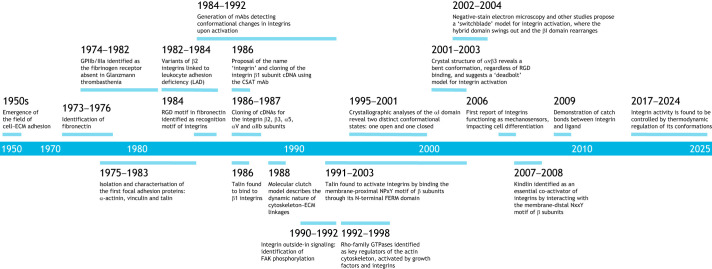
Historical timeline of the key discoveries in integrin research.

In this Perspective, we reflect on the evolution of the integrin field, highlighting the key early discoveries that have shaped its trajectory (Fig. 1). Whereas numerous comprehensive reviews by esteemed colleagues provide in-depth analyses of various aspects, our focus is necessarily selective. We recognise the valuable contributions of many researchers and regret any omissions necessitated by space constraints.

## The beginning

When we embarked on our integrin research, we first needed to familiarise ourselves with the fast-growing integrin literature, including a substantial body of research on related topics such as cell adhesion, cell locomotion and lamellipodia. The latter studies were published by pioneers such as Adam Curtis and Michael Abercrombie, who at the time could not have known they were studying what we now recognise as integrin-dependent processes. Curtis (1964) used interference reflection microscopy, developed in the 1950s, to demonstrate that several ventral plasma membrane patches in adhering amphibian cells are near the substrate, whereas the rest of the ventral membrane is further away. Although the quality of the images was not ideal, we now view these findings as the first description of adhesion plaques. In his 1964 paper, Curtis also demonstrated that ethylenediaminetetraacetic acid (EDTA) treatment increased the distance between the ventral membrane and the substrate, aligning with structural studies performed almost 30 years later, which showed that integrin–ligand binding is metal binding dependent (Curtis, 1964). Abercrombie and colleagues (Abercrombie et al., 1971) used electron microscopy to describe ‘adhesion plaques’ in the leading lamella, which they observed to be linked to ‘longitudinal filaments’. This marked the first description of actomyosin-containing stress fibres terminating at sites now recognised as focal adhesions – multiprotein structures in which integrins play a central role by linking the extracellular matrix (ECM) to the actin cytoskeleton.

## Identification of fibronectin

Although the papers published by Curtis and Abercrombie can be viewed as the starting point of focal adhesion research, the breakthrough discovery that paved the way for ‘integrin adhesion biology’ came from the identification of the integrin ligand fibronectin. Fibronectin was independently identified in 1973 by several researchers, including Hynes (1973), Gahmberg and Hakomori (1973), and Ruoslahti and colleagues (Ruoslahti et al., 1973). Hynes, as well as Gahmberg and Hakomori, were searching for cell surface proteins that differed quantitatively between non-transformed and virus-transformed cells. Hynes (1973) isolated ‘large external transformation sensitive protein (LETS)’, and Gahmberg and Hakomori (1973) isolated ‘galactoprotein a’. Both proteins were only detectable on non-transformed cells. Ruoslahti and colleagues (Ruoslahti et al., 1973) isolated ‘surface (SF) antigen’ from the surface of chicken embryo fibroblasts, raised an antiserum against it, and were the first to demonstrate that the SF antigen assembled into an elaborate, fibrillar network on the cell surface. The ability of these proteins to form such networks and support cell attachment led Vaheri and Ruoslahti, along with Richard Hynes and his team, to name this new ECM protein fibronectin (Hynes, 1990). They also showed that the SF antigen is present in serum and that fibronectin is the same molecule as the ‘cold-insoluble globulin’ in serum described a few years earlier (Mosesson and Umfleet, 1970).

Several laboratories later confirmed that fibronectin mediates adhesion, influences cell morphology and promotes cell migration. Immunostaining demonstrated that fibronectin is linked to actin, likely by ‘interacting with (cell) surface molecules’ (Hynes, 1976). However, at the time, a direct interaction between fibronectin and actin within the membrane or submembranous region was also considered (Hynes, 1976). These findings formed the foundations of the search for fibronectin-binding proteins on cells.

## Defining the integrin family using monoclonal antibodies and biochemical approaches

In the field of platelet research, many groups sought to understand how activated platelets bind fibrinogen during aggregation and clot formation. Although integrins had not yet been identified at the time, these studies made important contributions to the field of integrin biology. Notably, Glanzmann thrombasthenia, a genetic bleeding disorder characterised by prolonged bleeding time due to the inability of platelets to aggregate, provided important insights into the molecular mechanisms underlying platelet function and was instrumental in the discovery of platelet integrins and their role in cell adhesion. In two seminal papers, Nurden and Caen showed that two proteins with molecular masses of 135 kDa and 103 kDa (Nurden and Caen, 1974, 1975), named GPIIb (later named αIIb integrin) and GPIIIa (β3 integrin) (Phillips and Agin, 1977a,b), respectively, were absent in lysates from platelets isolated from individuals with Glanzmann thrombasthenia.

Marguerie and colleagues (Marguerie et al., 1979), along with Bennett and Vilaire (1979), demonstrated that platelets possess a surface receptor whose binding activity can be induced by ADP or epinephrine. Since Bennett and Vilaire also found that fibrinogen binding could not be induced in Glanzmann thrombasthenia platelets, they speculated that the surface proteins GPIIb and GPIIIa were candidate receptors for fibrinogen. Indeed, in a follow-up paper, Bennett and colleagues used chemical crosslinking to demonstrate that fibrinogen could be crosslinked to GPIIIa (Bennett et al., 1982). A breakthrough was published in the same year by Nachman and Leung (1982), who demonstrated that GPIIb and GPIIIa form a macromolecular 1:1 complex (GPIIb/IIIa), and that this complex, when purified from platelets, could directly bind purified fibrinogen. The latter notion was confirmed by Jennings and Phillips (1982), who demonstrated that GPIIb and GPIIIa co-elute during gel filtration chromatography and can be co-immunoprecipitated from platelets using an anti-GPIIb antibody.

Studies on immune cell adhesion also played a pivotal role in the discovery of integrins and in elucidating how these receptors are activated to mediate cell adhesion to the vessel wall. While in the lab of César Milstein (Cambridge, UK), Springer generated a mAb against an antigen present on differentiated macrophages, named Mac1 (Springer et al., 1979), which was later identified as the αM subunit of the αMβ2 integrin. Todd et al. (1981), along with Ceuppens et al. (1982), also generated mAbs, called Mo1 and OKM, respectively, and showed that these mAbs recognise the Mac1 antigen. After returning to the USA, Springer identified LFA-1 and the p150,95 molecule, revealing that LFA-1, Mac1 and p150,95 share a common β subunit, thereby defining the β2-class integrin subfamily (Sanchez-Madrid et al., 1983). Around the same time, Arnaout and colleagues (Arnaout et al., 1982), as well as Dana et al. (1984), reported that individuals carrying mutations in β2 integrin genes suffer from a leucocyte adhesion deficiency (LAD), which pointed to an essential role of integrins during leukocyte extravasation (Lawrence and Springer, 1991). Meanwhile, Hemler and colleagues used a panel of mAbs to identify a family of cell surface antigens, VLA1–5 (Hemler et al., 1985, 1987). The family was named after its first two members, VLA1 and VLA2, which are expressed very late during T cell activation. However, these proteins, including VLA3, VLA4 and VLA5, were later found to be broadly expressed across various cell types, not limited to lymphocytes (Kantor et al., 1987). They share a common β subunit and constitute the first five members of the β1-class integrins (VLA1, α1β1 integrin; VLA2, α2β1 integrin; VLA3, α3β1 integrin; VLA4, α4β1 integrin; and VLA5, α5β1 integrin).

In parallel, Wilcox and Brower used mAbs to identify cell surface antigens with position-specific expression in *Drosophila* imaginal discs (Brower et al., 1984). These position-specific antigens, PS1 and PS2, consist of distinct α subunits paired with a common β subunit and show a high degree of biochemical and structural homology to vertebrate integrins.

## Cloning of cell substrate attachment antigen, isolation of RGD-binding receptors, and defining the β1-, β2- and β3-class integrins

Two key ‘integrin papers’ reported the generation of anti-chicken mAbs, called JG22 and cell substrate attachment (CSAT) (Greve and Gottlieb, 1982; Neff et al., 1982), that detached chicken cells from different ECM proteins, including collagen, fibronectin and laminin. Particularly informative was the work with the CSAT mAb produced by the Horwitz group (Neff et al., 1982), which labelled adhesion plaques in adherent fibroblasts that partially overlapped with actomyosin stress fibres (Chen et al., 1985; Damsky et al., 1985). This mAb enabled the isolation of glycoproteins with molecular masses of 120 kDa (later identified as the β1 subunit) and 160 kDa (co-purified α subunits), as well as the cloning of the first integrin subunit cDNA by the Hynes group (Tamkun et al., 1986). The cDNA clone encoding the CSAT antigen produced a polypeptide of 89 kDa. The protein complex containing this 89 kDa polypeptide was named ‘integrin’ to reflect the functional properties of the CSAT antigen as an ‘integral membrane protein complex linking the extracellular matrix with the cytoskeleton’. A year later, cloning of the β2 integrin subunit (Kishimoto et al., 1987; Law et al., 1987) and the GPIIIa (β3 integrin) subunit (Fitzgerald et al., 1987) confirmed their similarity to the gene encoding the β1 integrin identified using the CSAT mAb.

Around the same time that the Hynes group cloned and sequenced the CSAT antigen, Ruoslahti and colleagues took a different but equally successful approach to clone integrin cDNAs by building on the isolation and characterisation of the receptors. First, Pierschbacher and Ruoslahti reported in their groundbreaking paper (Pierschbacher and Ruoslahti, 1984) that the Arg-Gly-Asp-Ser (RGDS) tetrapeptide of fibronectin is sufficient to mediate cell attachment and to inhibit attachment of cells to fibronectin. Following the identification of this minimal cell attachment peptide, the Ruoslahti group used the RGDS sequence with an additional proline residue at the C terminus to isolate a fibronectin-binding protein complex (Pytela et al., 1985b) (later identified as α5β1 integrin) and a vitronectin-binding protein complex (Pytela et al., 1985a) (later identified as αVβ3 integrin). The Carter group was the first to report the presence of an RGD-independent integrin-binding site in the alternatively spliced type III connecting segment (IIICS) of fibronectin that specifically binds to VLA4 (α4β1 integrin; Wayner et al., 1989). By using the purified fibronectin receptor as an immunogen, Ruoslahti and colleagues generated antibodies that allowed them to isolate the cDNAs for the α5 and β1 subunits (Argraves et al., 1986, 1987), and antibodies against the vitronectin receptor facilitated cloning of the αV subunit cDNA (Suzuki et al., 1987). The Bennett group cloned the GPIIb (αIIb) subunit and recognised its high homology to the α5 and αV subunits (Argraves et al., 1986, 1987; Poncz et al., 1987).

In the same time frame that the vertebrate integrin subunits were cloned, cDNAs for the *Drosophila* PS2α and PS2β subunits were also isolated (Bogaert et al., 1987; MacKrell et al., 1988). The cloning of these integrin subunits established integrins as an evolutionarily conserved protein family, even though the precise mechanisms underlying their functions still remained unknown.

## Identification of the first fibronectin–integrin adhesome proteins

Around the same time that integrin subunits were cloned, Keith Burridge and Benny Geiger pioneered the isolation and characterisation of the first focal adhesion proteins. The Burridge group (Lazarides and Burridge, 1975) prepared antibodies against muscle α-actinin, which had already been isolated in 1964 by Ebashi and Ebashi (Ebashi and Ebashi, 1964), and immunostained adherent non-muscle cells. They observed periodic immunosignals along filament bundles (which they correctly assumed to be actin) and scattered, patchy signals (which they correctly assumed to be adhesion plaques) where groups of filament bundles terminate (Lazarides and Burridge, 1975).

Geiger (1979) used a clever protein extraction method to isolate and purify a 130 kDa protein from chicken gizzard, now known as vinculin. This protein was found predominantly in two intracellular regions: in cell–substrate adhesion plaques at the termini of actin filament bundles and in cell–cell contacts. A few months later, the Burridge group (Burridge and Feramisco, 1980) isolated the same 130 kDa protein and observed its presence in adhesion plaques that assemble at the termini of actin bundles at the ventral plasma membrane. Additionally, they found that the protein localised with the fibrillar fibronectin network on the dorsal cell surface. They suggested that a surface receptor probably links the intracellular 130 kDa protein to extracellular fibronectin and might ‘utilise this association for a motile function by pulling on the 130 kDa protein’ (Burridge and Feramisco, 1980). This was a remarkably farsighted conclusion.

The third focal adhesion protein discovered after α-actinin and vinculin was talin, a 215 kDa protein isolated from chicken gizzard by the Burridge group (Burridge and Connell, 1983). The isolation of talin was crucial for understanding how integrins function. Like vinculin – later shown to bind talin through 11 vinculin-binding sites buried in 13 helical bundles – talin is found in adhesion plaques at the ends of actin filaments and in ruffling membranes, and it colocalises with fibronectin fibrils on the cell surface. Following the discovery of talin, the Horwitz and Burridge groups (Horwitz et al., 1986) demonstrated direct binding between talin and the CSAT antigen. After this milestone paper, the Ginsberg group highlighted the involvement of talin in integrin–ligand binding activity, and the Springer group described the role of talin in transducing cytoskeletal forces to stabilise the activated conformation of integrins (see below).

In the following years, numerous focal adhesion proteins were discovered. Although it is impossible to mention all of them, a few notable examples, including the focal adhesion kinase (FAK, also known as PTK2) and p130Cas (also known as BCAR1), will be discussed below in the section ‘Integrin outside-in signalling’. A first collection of adhesome proteins, defined by their localisation and/or their ability to regulate adhesion complexes, has been compiled by mining the literature (Zaidel-Bar et al., 2007). This list has subsequently been expanded by the isolation and analysis of the adhesome of suspended cells (Humphries et al., 2009) and adherent cells (Kuo et al., 2011; Schiller et al., 2011).

## Integrin activation: conformational changes, talin and kindlin

The first hints that integrins require an ‘activation’ step to bind ligands were obtained from studies on fibrinogen binding to platelets. As mentioned above, resting platelets have low binding activity for soluble fibrinogen, whereas activated platelets bind fibrinogen and subsequently aggregate. One of the major unsolved problems concerning platelet aggregation was understanding how the receptor function of the GPIIb/IIIa complex (αIIbβ3 integrin) is activated by agonists such as ADP, thrombin and collagen. Since the number of GPIIb/IIIa complexes on the platelet surface increases only slightly upon platelet stimulation (due to the fusion of secretory α-granules containing GPIIb/IIIa), it was assumed that the GPIIb/IIIa activation response occurs through the activation of pre-existing surface receptors (Bennett and Vilaire, 1979; Marguerie et al., 1979). This hypothesis was confirmed using mAbs that were shown to bind activation-induced epitopes on GPIIb/IIIa (Coller, 1985; Kouns and Jennings, 1991; McEver and Martin, 1984; Shattil et al., 1985), as well as by fluorescence resonance energy transfer (FRET) induced during platelet activation between fluorescently labelled antibodies bound to epitopes in the GPIIb and GPIIIa ectodomains (Sims et al., 1991).

In the early 1990s, several reports demonstrated that conformational changes defining different affinity states for ligands also occur in β2- and β1-class integrins. Van Kooyk et al. (1991) generated the mAb NKI-L16. This mAb recognises a neoepitope on LFA-1 that promotes lymphocyte adhesion upon induction with the cell adhesion regulator phorbol diester phorbol 12-myristate 13-acetate (PMA). Kovach et al. (1992) produced the mAb 8A2, which promotes VLA4 (α4β1), VLA5 (α5β1) and VLA6 (α6β1) integrin-mediated adhesion of peripheral blood lymphocytes to VCAM-1, fibronectin and laminin, respectively. The Ginsberg group (Faull et al., 1993) reported that mAb 8A2 induces a neoepitope on the β1 subunit that allows VLA5 to bind fibronectin in a signalling-independent manner. These studies confirmed an earlier report by Shimizu and colleagues (Shimizu et al., 1990), which showed that lymphocytes bind strongly to fibronectin via VLA4 (α4β1) and VLA5 (α5β1), and to laminin via VLA6 (α6β1), but only after cell activation. Taken together, these papers, along with many others, suggested that a conformational change is induced in ‘activated’ integrins, that this conformational change is required for ligand binding and cell adhesion, and that this process is not limited to blood cells.

Several groups, most prominently the Ginsberg group, manipulated the short α- and β-integrin cytoplasmic tails and reported that deletion of either tail constitutively activates integrins, whereas truncations of the more C-terminal region prevent activation (O'Toole et al., 1991). It was concluded that the membrane-proximal regions of the α- and β-integrin tails interact and that this interaction is broken during activation (O'Toole et al., 1991). This hypothesis was subsequently confirmed when the Ginsberg group identified a salt bridge between αIIb and β3 integrin tails, located close to the inner side of the plasma membrane, which locks the integrin in a low-affinity conformation (Hughes et al., 1996). Additionally, this salt bridge, along with several hydrophobic interactions between the membrane-proximal α helices of the αIIb and β3 integrin subunits, was observed in the nuclear magnetic resonance structure of the αIIbβ3 cytoplasmic complex determined by the Qin and Plow groups (Ma et al., 2006). The Springer group published FRET experiments confirming that integrin tail separation also occurs in cells upon integrin activation or ligand binding (Kim et al., 2003).

O'Toole and colleagues were the first to observe that the membrane-proximal NPxY motif plays a key role in regulating integrin affinity conformation (O'Toole et al., 1995). A breakthrough finding published by the Ginsberg group revealed that talin binds via its N-terminal four-point-one, ezrin, radixin and moesin (FERM) domain to the cytoplasmic tails of the β1 integrin isoforms β1A and β1D (a muscle-specific splice variant) and of β3 integrin (Calderwood et al., 1999). This association with talin enhances ligand binding and increases binding of the activation epitope-recognising mAb PAC1 to αIIbβ3 expressed in CHO cells in an NPxY-dependent manner (Calderwood et al., 1999; Tadokoro et al., 2003). Structural studies showed that the F3 subdomain of the talin FERM domain resembles a phosphotyrosine-binding domain and binds the membrane-proximal NPxY motif of β integrin tails (Calderwood et al., 2002; Garcia-Alvarez et al., 2003).

These and other studies provided the basis for concluding that talin acts as ‘a common final step in integrin activation’ (Calderwood et al., 1999; Tadokoro et al., 2003). However, subsequent research by the Wu group revealed that, in addition to talin, a protein initially called MIG2, later renamed kindlin-2 (also known as FERMT2), also binds β integrin tails and activates integrins when overexpressed in CHO cells (Shi et al., 2007). This function of kindlin proteins is evolutionary conserved, as demonstrated by studies in *Caenorhabditis elegans*, where the kindlin homologue UNC-112 is essential for integrin-mediated muscle attachment (Rogalski et al., 2000). In mammals, the kindlin family comprises three members (kindlin-1, -2 and -3), whose roles in integrin activation have been confirmed in mouse genetic studies as well as cell-based experiments (Ma et al., 2008; Moser et al., 2008). Loss of kindlin-1 expression in humans leads to a skin blister disease, whereas loss of kindlin-3 causes LAD type III, a rare syndrome characterised by severe bleeding and leukocyte adhesion deficiency (Moser et al., 2009).

## Integrin activation: conformational changes – from the αI domain to the entire integrin molecule

Understanding the integrin activation process began with a major breakthrough: the crystallisation of integrin αI domains. The αI domain is a subdomain of ∼190 amino acids that binds ligands. It is found inserted (hence ‘I domain’) in the head domain of nine out of the 18 known α subunits. The remaining nine α subunits lack an αI-domain. Structural studies by the Liddington group (Lee et al., 1995b) revealed that the αI domain of Mac1 adopts a Rossmann fold, characterised by central β sheets flanked by α helices. This domain also features a metal-binding site called metal ion-dependent adhesion site (MIDAS), which plays a crucial role in coordinating ligand and divalent cation binding. The Liddington group predicted that a similar MIDAS exists within the integrin β subunit, a hypothesis later confirmed when the first structure of an integrin ectodomain was solved (Xiong et al., 2001). Further structural studies by Lee et al. (1995a) and Qu and Leahy (1995) revealed that the αI domain of Mac1 and LFA-1, respectively, can adopt two distinct conformations: an open (high-affinity, liganded) and a closed (low-affinity, unliganded) state. These conformational states are regulated by metal binding and associated with large conformational changes across the entire protein domain. These were the first conformational changes observed in an integrin domain structure that regulate ligand affinity. These important observations were subsequently confirmed by the Liddington group, who determined the structures of the αI domain from the collagen-binding α2β1 integrin in both its occupied state (with collagen-like peptides) and unoccupied state (Emsley et al., 2000). Several more structures, not discussed here, further corroborated these groundbreaking observations.

Two seminal papers from the Arnaout group marked the next significant advance. They reported the structures of the αVβ3 integrin ectodomain (which lacks an αI domain) in both its occupied state (bound to a cyclo-RGDF peptide) and its unoccupied state (Xiong et al., 2001, 2002). These structures clarified the domain composition of α- and β-subunits, confirmed the prediction by the Liddington group (Lee et al., 1995b) that the β subunit adopts an I domain-like fold with a MIDAS, and demonstrated that the βI domain inserts into the hybrid domain. The studies also revealed how the βI domain associates with the seven-bladed β-propeller domain of the α subunit, forming the ligand-binding pocket in integrins without I domains.

The most surprising discovery from the structures was that the ‘legs’ of the α- and β-subunits were bent at the ‘knee’, regardless of whether the integrin was bound to a ligand or not (Xiong et al., 2001, 2002). This was particularly notable because the Springer group, after studying the ligand-bound integrin ectodomain, had proposed that knee extension is necessary for integrin activation. Their conclusion was based on observations made using negative-stain electron microscopy, which revealed that the integrin ectodomain adopts three distinct conformations: a bent conformation, where the binding pocket faces the plasma membrane, and two extended conformations, one with a closed (unliganded) and one with an open (liganded) headpiece (Takagi et al., 2002). They suggested a ‘switchblade-like’ mechanism to explain the transition from bent to extended conformation.

The Arnaout group responded by proposing that knee extension might occur as ‘a post-binding event linked to outside-in signalling’. They argued that activation is controlled by a loop in the lower leg domain, which acts as a deadbolt to lock or unlock the βI domain. This became known as the ‘deadbolt hypothesis’ (Xiong et al., 2003). However, further studies from the Springer group refuted this hypothesis (Zhu et al., 2007).

The extended-open conformation was found to be accompanied by integrin leg separation, as predicted by the Ginsberg group (Hughes et al., 1996) and confirmed by the Qin group (Vinogradova et al., 2002). This also involved an outward swing of the hybrid domain away from the α subunit. This shift in the hybrid domain triggers conformational changes in the βI domain (Takagi et al., 2002), similar to those described earlier for αI domains by the Liddington group (Emsley et al., 2000). These findings, along with numerous follow-up papers, established the switchblade mechanism linked with hybrid domain swing-out and βI domain rearrangements as the activation model for 22 out of 24 integrins (Xiao et al., 2004).

Additionally, the Springer lab solved the structure of the complete ectodomain of the αI domain-containing αXβ2 integrin, which revealed how conformational changes in the βI domain are communicated to the αI domain to open the ligand-binding site (Nishida et al., 2006). Since the publication of these structures, several groups have employed various structural and biochemical methods, including cryo-electron microscopy, to confirm the switchblade model and further refine the mechanism underlying integrin activation.

## Integrin inside-out signalling and the thermodynamic model

The ability of integrins to regulate their affinity for ligands is known as integrin activation–deactivation. As mentioned above, this fascinating process was first observed in platelets. When G-protein-coupled receptors on the surface of platelets bind agonists like thrombin, ADP or chemokines, they trigger an intracellular signalling pathway that activates the fibrinogen-binding integrin. This pathway is referred to as ‘inside-out signalling’.

Based on their observations that talin binds to β integrin cytoplasmic tails and unclasps the α- and β-integrin cytoplasmic tails and their transmembrane domains (TMDs) (Calderwood et al., 1999; O'Toole et al., 1991), the Ginsberg group proposed (Ginsberg, 2014) that talin-bound β integrin cytoplasmic tails tilt the β integrin TMD, inducing extension of the integrin ectodomain and opening of the ligand-binding domain. The conclusion that talin binding to β integrin cytoplasmic tails is sufficient for monomeric integrin activation was supported by experiments showing activation of purified full-length integrins embedded in lipid membranes by the N-terminal talin-FERM domain (Ye et al., 2010). However, it is important to note that the increase in integrin activation was relatively modest, with only a twofold increase in PAC1 mAb binding. This raised the question of whether other factors, such as kindlins, are also involved in integrin activation or if the entire model needs to be revised. It was subsequently confirmed that kindlin is indeed an essential integrin co-activator (Moser et al., 2009). Furthermore, the Springer group demonstrated in a series of excellent papers that thermodynamics govern the equilibrium between different integrin conformations, with the active, extended-open conformation stabilised by ligand binding and tensile forces transmitted to the liganded integrins via talin (or kindlin) (Li et al., 2024; Li and Springer, 2017). Recently, the question of how the low-affinity binding of talin to the β integrin cytoplasmic tail (300–500 µM) resists actin cytoskeleton-generated forces was investigated. It was found that kindlins augment the low affinity for talin by inducing a conformational change in the β integrin tail structure, which in turn allows the propagation of force to the integrin–ligand bond (Aretz et al., 2023; Bodescu et al., 2023).

## Integrin mechanotransduction at focal adhesions

The forces required to stabilise the integrin–ligand bond are generated by the actomyosin cytoskeleton. Small GTPases, which are key regulators of actomyosin assembly and contractility, play a crucial role in this process. These GTPases became central to the integrin field following the seminal discoveries by Ridley, Hall and colleagues (Ridley and Hall, 1992; Ridley et al., 1992). These authors showed that growth factors (and later, integrins themselves) activate RhoA to promote the assembly of focal adhesions and contractile stress fibres, whereas Rac1 activation leads to membrane ruffling. Subsequently, several groups reported that myosin II-driven contractility of stress fibres and actin polymerisation, both essential for the assembly and function of focal adhesions, are mediated by the RhoA targets Rho-associated kinase (ROCK, collectively referring to ROCK1 and ROCK2) and the formin mDia (also known as DIAPH1), respectively.

Although many studies have investigated integrin-mediated mechanotransduction, several stand out as particularly influential. The first was by Bershadsky and colleagues (Riveline et al., 2001), who showed that mechanically stretching the periphery of an adherent cell with a pipette induces a RhoA–ROCK–mDia-dependent, streak-like enlargement of integrin-based adhesion sites. A seminal study from the Discher group (Engler et al., 2006) demonstrated that mesenchymal stem cells sense matrix stiffness through integrin-mediated adhesions and, in conjunction with growth factor signals, direct their lineage differentiation. A further set of studies by the Sheetz and Garcia groups showed that mechanical unfolding of talin promotes vinculin recruitment, and that vinculin turnover at focal adhesions decreases as force increases (Del Rio et al., 2009; Dumbauld et al., 2013). Finally, the Waterman group demonstrated that focal adhesions are organised in functional layers: a force-transducing layer, made up of proteins like talin and vinculin, is positioned just beneath the layer where integrins transmit signals into the cell (Kanchanawong et al., 2010).

A further key paper was published by the Zhu group (Kong et al., 2009), demonstrating that the magnitude of force applied to the integrin–ligand bond directly influences bond lifetime. They found that increasing the pulling force on the bond results in a longer bond lifetime. This phenomenon, known as catch bond formation, is driven by force-induced conformational changes in the integrin (a process referred to as mechanical allostery). Although the precise structural details of this conformational change remain unresolved, it strengthens the bond between integrin and ligand. This bond is distinct from typical protein interactions known as slip bonds, which weaken and separate under force rather than strengthen.

The molecular clutch model described by Mitchison and Kirschner (1988) also profoundly influenced the integrin community, enhancing our understanding of how integrins convert mechanics (physics) into biochemical signals (chemistry). According to the model, forces generated by actin contractility, coupled with actin filament polymerisation activity pushing against the plasma membrane, produce a backward movement of the actin flow called ‘actin retrograde flow’. The model further predicts that anchorage of actin filaments through integrins and associated proteins (such as talin and vinculin) to the immobile ECM opposes actomyosin contractility and retrograde actin flow. This interaction leads to conformational changes in talin (opening of vinculin-binding sites), tyrosine phosphorylation of p130Cas, protrusion of the leading-edge membrane and strengthening of integrin–ligand bonds. Thus, the molecular clutch provides an efficient mechanism that allows cells to dynamically adjust and balance tissue mechanics during processes such as development, wound healing and cancer progression.

## Integrin outside-in signalling

The activation of biochemical signalling cascades in cells occurs through the adhesome – a network of proteins located at adhesion sites (Zaidel-Bar et al., 2007). Early cell adhesion research showed that adaptor proteins and protein tyrosine kinases play key roles in focal adhesion assembly and signalling. Two examples that received broad interests are described here.

Immunostaining of adherent cells with non-specific anti-phosphotyrosine antibodies was found to prominently label focal adhesions (Maher et al., 1985; Marchisio et al., 1984). Additionally, transformation of cells by oncogenic v-Src was observed to decrease cell adhesion, alter cell morphology and increase phosphorylation of focal adhesion proteins, particularly paxillin and the then newly identified focal adhesion-associated protein tyrosine kinase, later termed FAK. FAK was originally discovered by the Parsons group (Kanner et al., 1990; Schaller et al., 1992) and is likely the same protein that the Hynes (Guan et al., 1991) and Juliano groups (Kornberg et al., 1991) had previously shown to become phosphorylated upon cell adhesion to fibronectin and upon antibody-mediated clustering of β1 and α3 integrin subunits, respectively. The Brugge and Parsons groups (Lipfert et al., 1992) demonstrated that FAK phosphorylation is induced in activated platelets but not in platelets isolated from patients suffering from Glanzmann thrombasthenia, providing genetic proof that FAK phosphorylation occurs downstream of integrin signalling.

Around the same time, the Hanafusa group (Matsuda et al., 1990) identified several phosphotyrosine-containing proteins, including a 130 kDa protein, that interacted with the oncogenic product P47*^gag-crk^* of the avian sarcoma virus CT10. The Hirai group (Sakai et al., 1994) subsequently reported that v-Crk and v-Src can form a ternary complex with a 125–130 kDa protein (p130), which they named p130Cas (for Crk-associated substrate). Polte and Hanks (1995) showed that FAK can bind and phosphorylate p130Cas. Subsequent findings revealed that the FAK–p130Cas complex can activate the small GTPases Rac1 and Rap1 in a force-dependent manner. The force-dependent activation of Rap1 requires Src-mediated phosphorylation of p130Cas, as elegantly demonstrated by the Sheetz group (Sawada et al., 2006) using *in vitro* stretching experiments of recombinant p130Cas in the presence of recombinant v-Src.

## Genetic analysis and integrins as pharmaceutical targets

The ablation of genes encoding the integrin α- and β-subunits in mice (Bouvard et al., 2001) not only confirmed known integrin functions but also unveiled novel roles, identified specific integrins as novel disease-causing genes and clarified their involvement in various diseases. These studies subsequently led to the development of novel drugs with applications in conditions such as lung diseases (chronic obstructive pulmonary disease and asthma; see ‘Therapeutic Insights from Integrin Knockout Mice’, https://www.youtube.com/watch?v=VmFOKSpngPg), Morbus Crohn (Crohn's disease) and multiple sclerosis (MS).

A compelling example of the therapeutic potential of targeting integrins is MS. Yednock and colleagues (Yednock et al., 1992) first demonstrated the involvement of integrins in MS by showing that treatment of a MS mouse model with blocking anti-α4β1 integrin antibodies prevents autoreactive T cell extravasation and disease onset. Building on this discovery, companies developed the humanised anti-α4β1 antibody natalizumab (Tysabri**^®^**), which reduced brain lesions and the frequency of relapses in patients suffering from relapsing–remitting MS (Tubridy et al., 1999). Natalizumab treatment of MS patients was approved by the US Food and Drug Administration despite the rare risk of developing progressive multifocal leukoencephalopathy (PML). PML is a fatal opportunistic infection caused by the reactivation of a latent John Cunningham virus (JC virus), which leads to severe brain damage and can occur in immunodeficient or immunosuppressed patients.

## Conclusion and future perspectives

The advent of hybridoma technology, which enables researchers to generate highly specific mAbs, along with the development of novel expression cloning techniques, was instrumental in identifying and characterising the integrin family. These technological advances revealed that various molecules that were previously regarded as distinct entities are, in fact, structurally and functionally related, indicating shared roles in key cellular processes. Subsequently, genetics linked integrins to crucial physiological processes such as platelet aggregation, leukocyte extravasation, development and disease progression.

A key lesson from the integrin discovery journey is that a strong spirit of collaboration was essential. The collaborative environment that fuelled progress was exemplified by the open and collegial exchange of unpublished data and crucial reagents, such as the CSAT antibody, which enabled the Hynes lab to clone the CSAT antigen (Tamkun et al., 1986), the first integrin identified. Furthermore, fibronectin – rather than other ECM components such as collagen (discovered around 1870) or laminin (identified shortly after fibronectin) – played a pivotal role in the discovery of ECM-binding integrins. Fibronectin is a single-chain protein that could be readily expressed and isolated from plasma and contains a minimal integrin-binding motif, RGD. In contrast, collagens and laminin are complex, multichain proteins that are difficult to express. In the case of laminin, a mysterious 67 kDa protein was initially thought to be its cell surface receptor, until the groups of Ruoslahti and Sonnenberg showed in 1988 that laminin binds to integrins α3β1 and α6β1, respectively (Gehlsen et al., 1988, 1989; Sonnenberg et al., 1988). The earliest evidence of a collagen receptor belonging to the integrin family likely came from studying an individual with a defective response to collagen, traced to the absence of GPIa, now identified as the α2 integrin subunit. (Nieuwenhuis et al., 1985; Santoro, 1986).

Although integrin biology is now broadly understood, many crucial details remain unresolved. These include the dynamic regulation of integrin affinity by intracellular and extracellular signals; the molecular mechanisms by which integrin signalling influences cell proliferation, migration and differentiation; and the endocytic and exocytic trafficking of integrins during focal adhesion remodelling. The roles of the α subunit cytoplasmic domains, including their alternatively spliced variants, in integrin function has yet to be fully elucidated.

A major limitation in current research is the reliance on two-dimensional model systems, which often fail to capture the complexity of integrin-mediated interactions *in vivo*. There is a pressing need for experimental models that more accurately reflect physiological conditions. Another crucial gap lies in the incomplete understanding of the ‘adhesome’ – including the posttranslational modifications occurring before and after integrin ligation, and how these modifications regulate the broader adhesome machinery. Furthermore, the roles of integrin adhesion in development and postnatal homeostasis, and the involvement of integrins in epithelial cell polarity, crosstalk with cell–cell adhesion and transduction of growth factor signals still remain poorly understood.

Celebrating 100 years of The Company of Biologists
2025 marks 100 years since the formation of The Company of Biologists. As part of our celebrations, we are sharing content about the past, present and future of the Company and reaching out to extraordinary members of our community to bring you new and original material.

Arnoud Sonnenberg is a Retired Group Leader at the Netherlands Cancer Institute, Amsterdam, and Professor Emeritus of Cell Adhesion in Human Health and Disease at Leiden University, the Netherlands. His lab investigates the function of integrins in differentiation and migration, and how they regulate the assembly of multiprotein complexes in normal and pathological conditions. Arnoud also served as an Editor for Journal of Cell Science from 2005 to 2023, dedicating his expertise and many years of service to this journal.

Reinhard Fässler is Director Emeritus of the Max Planck Institute of Biochemistry, Martinsried, and a Distinguished Affiliated Professor at the Technical University Munich, Garching, Germany. His lab studies how integrins are activated and carry out their signalling functions, why they recycle and how damaged integrins are recognised in endosomes. Over the years, Reinhard has been a regular contributor to Journal of Cell Science.
